# Measuring Regional Quality of Health Care Using Unsolicited Online Data: Text Analysis Study

**DOI:** 10.2196/13053

**Published:** 2019-12-16

**Authors:** Roy Johannus Petrus Hendrikx, Hanneke Wil-Trees Drewes, Marieke Spreeuwenberg, Dirk Ruwaard, Caroline Baan

**Affiliations:** 1 Tranzo Scientific Center for Care and Welfare Tilburg University Tilburg Netherlands; 2 Center for Nutrition, Prevention and Health Services National Institute for Public Health and the Environment Bilthoven Netherlands; 3 Zuyd University of Applied Sciences Heerlen Netherlands; 4 Department of Health Services Research, Care and Public Health Research Institute Faculty of Health, Medicine and Life Sciences Maastricht University Maastricht Netherlands

**Keywords:** text mining, population health management, regional care, quality of care, online data, big data, patient-reported experience measures

## Abstract

**Background:**

Regional population management (PM) health initiatives require insight into experienced quality of care at the regional level. Unsolicited online provider ratings have shown potential for this use. This study explored the addition of comments accompanying unsolicited online ratings to regional analyses.

**Objective:**

The goal was to create additional insight for each PM initiative as well as overall comparisons between these initiatives by attempting to determine the reasoning and rationale behind a rating.

**Methods:**

The Dutch Zorgkaart database provided the unsolicited ratings from 2008 to 2017 for the analyses. All ratings included both quantitative ratings as well as qualitative text comments. Nine PM regions were used to aggregate ratings geographically. Sentiment analyses were performed by categorizing ratings into negative, neutral, and positive ratings. Per category, as well as per PM initiative, word frequencies (ie, unigrams and bigrams) were explored. Machine learning—naïve Bayes and random forest models—was applied to identify the most important predictors for rating overall sentiment and for identifying PM initiatives.

**Results:**

A total of 449,263 unsolicited ratings were available in the Zorgkaart database: 303,930 positive ratings, 97,739 neutral ratings, and 47,592 negative ratings. Bigrams illustrated that feeling like not being “taken seriously” was the dominant bigram in negative ratings, while bigrams in positive ratings were mostly related to listening, explaining, and perceived knowledge. Comparing bigrams between PM initiatives showed a lot of overlap but several differences were identified. Machine learning was able to predict sentiments of comments but was unable to distinguish between specific PM initiatives.

**Conclusions:**

Adding information from text comments that accompany online ratings to regional evaluations provides insight for PM initiatives into the underlying reasons for ratings. Text comments provide useful overarching information for health care policy makers but due to a lot of overlap, they add little region-specific information. Specific outliers for some PM initiatives are insightful.

## Introduction

With respect to evaluating experienced quality of care, unsolicited online ratings given to health care providers have received more and more attention. This is a shift away from a past focus on solicited surveys. Studies have shown the potential of unsolicited data as a valuable resource to provide insight into the quality of care experienced at the provider level [1-3]. Furthermore, online data have some very interesting properties for policy makers and researchers, as they tend to be easier to collect, have a bigger reach, are generally cheaper, are consistently updated, and can consist of more responses than solicited surveys [1,4].

Insight into how experienced quality of care can be improved is a pivotal challenge for population management (PM) initiatives. The rising costs, changing care demands, and issues with the provided quality of care are pushing policy makers to take new approaches. Instead of health care being a reactive system based on individual demands, it should be a proactive system organized around a population’s needs [5,6]. This requires a whole-system approach in order to improve quality and efficiency, including prevention. As a result, reforms designated as population health management are becoming more and more widespread in health policy. Even though different definitions exist [7], PM initiatives generally focus on the health needs of a specified population across the continuum of health and well-being by introducing multiple interventions that organize services related to health and social care, as well as prevention and welfare [7,8]. PM initiatives often strive to achieve the Triple Aim by shifting focus from individuals to populations and by integrating care across health and social domains [8,9]. The Triple Aim was introduced by Berwick et al in 2008 and requires the simultaneous pursuit of improving population health and experienced quality of care, while reducing costs [10]. Examples of PM initiatives include the American Accountable Care Organizations [11], the National Health Service’s Vanguard sites [12], and the Dutch pioneer sites [13]. For the pursuit of the Triple Aim by such initiatives to be successful, each of the Triple Aim’s three pillars needs to be evaluated at the population or often regional level. Unsolicited online data could be a valuable source for evaluating the experienced-quality-of-care pillar. However, a previous study, utilizing the same dataset used in this study, explored rating distributions and applied multilevel analyses. Results from these analyses suggested that when using only the available quantitative data, their use at the regional level is limited [14]. First, while differences in mean ratings between providers were caused by differences in provider-specific characteristics, regional differences could not be attributed to differences in regional characteristics. This means that any variation in mean rating between regions does not point to a structural difference in, for example, quality of care or population. Second, no insight was provided regarding the reasoning behind any given rating and why it was either negative or positive. Additional methods and/or data are needed to make unsolicited data more valuable for regional initiatives.

Text comments could be able to provide a solution for the lack of regional specificity and reasoning of unsolicited provider ratings. Much of the created online data comes in the form of text; examples include tweets, Facebook posts, forum comments, and others. In health care, most rating websites provide patients with the opportunity to add comments to their ratings as well. Comments are already used for, among other things, competitive analyses and consumer sentiment analyses [1,15]. Combining ratings with their comments in analyses can provide insight into the reasoning and rationale behind a positive or negative rating [16,17]. Typically, interviews would have to be conducted to determine reasoning. However, at the population scale, conducting interviews is a costly and time-consuming endeavor and unsolicited data could significantly help in this regard. Despite the potential, adding comments to the accompanying unsolicited provider ratings when evaluating differences in experienced quality of care between regional initiatives has not yet been explored.

This study explores whether adding text comments—that accompany ratings—to regional analysis can provide additional insight into evaluating experienced quality of care. The goal is to determine the comments’ value for PM initiatives individually as well as when comparing initiatives. The largest health care ratings website in the Netherlands will be studied using different sentiment and machine learning analyses.

## Methods

### Dataset

The Zorgkaart Nederland website [18] provided the unsolicited online patient ratings. On this website, patients can both give and see reviews. To add a review, patients first select a care provider, which can be a care professional, such as a specific general practitioner or specialist, or an organization, such as a hospital department or nursing home. Quantitative data included ratings for six quality-of-care dimensions. These ranged from 1 to 10, with 1 indicating the worst experience and 10 the best. The six rated dimensions differed depending on the category of provider that is selected. For most providers, the dimensions were appointments, accommodation, employees, listening, information, and treatment. Qualitative data was gathered using a textbox where patients could elaborate on their ratings and add other relevant comments as well as the condition they were treated for. No further personal information about respondents was requested, but time stamps and email addresses were registered. Multimedia Appendix 1 shows a screenshot (Dutch) of the rating form from the Zorgkaart Nederland website (Figure A1.1). The Zorgkaart Nederland staff checked each submission for repeated entries, integrality, and anomalies, and gave an identifier to each one.

### Regions

Ratings and providers were clustered at the regional level using nine PM initiatives’ zip codes. These nine initiatives were selected in 2013 by the Dutch Minister of Health and are specified geographical areas in which different organizations cooperate to achieve the Triple Aim. They are spread out across the Netherlands and around 2 million people live in these regions in total. The Dutch National Institute for Public Health and the Environment was assigned their evaluation and set up the National Population Management Monitor for this purpose [13].

### Preprocessing

An Excel file was provided by the Dutch Patient Federation (DPF), meaning no Web scraping or duplicate removal was necessary. The dataset is available from the DPF upon request. Mean ratings were calculated for each entry by averaging the six ratings provided. This combination was proven to provide an approximation of overall quality of care for an entry [19]. This mean rating was also used to assign a sentiment to each rating based on the Net Promotor Score (NPS). This is an instrument that determines consumer loyalty and whether a consumer is a promotor or a detractor for a company; sentiments are scored as follows: <6.5=negative, ≥6.5 and <8.5=neutral, and ≥8.5=positive [20]. Furthermore, providers in the Zorgkaart data were grouped into the following categories: hospital care, nursing home, general practitioner, insurer, birth care, pharmacy, physiotherapy, youth care, dental care, and *other* (see Multimedia Appendix 1, Table A1.1).

Text comments were transformed into a so-called “bag-of-words,” which is required by the analyses described below. “Bag-of-words” means that any grammar, including punctuation, numbers, and capitalization, as well as word orders are removed from the text [21]. When the grammar is stripped away from a comment, that comment is then transformed into a matrix. In this matrix, each word (ie, unigram) or combination of two words (ie, bigram) is its own column. The rows are then filled with the number of times a word appears in that particular comment. This is done for all comments and creates a large matrix in which all comments and words in the whole dataset appear individually on the rows and columns. To tailor bigrams and prevent some word combinations from appearing positive, the previous words were evaluated and added if there were words such as “not” (“niet” in Dutch). For example “taken seriously” becomes “not taken seriously,” essentially creating a trigram in these cases. To further prepare the dataset, stop words (eg, “and,” “the,” and “with”) were removed. Words with a sparsity above 99% were also removed; this meant that these words only appeared in 1% or less of the comments, as it was expected that these words would not appear enough to be relevant for analyses. The dataset was transformed into a long or wide form, depending on the needs of the analyses. Finally, sentiment was established using two methods. First, the mean rating belonging to a comment was used to establish a positive, neutral, or negative sentiment to that comment (row). These categories were based on the NPS, as described above. Second, a Dutch lexicon was used to assign a polarity to each word (column) individually in the dataset [22]. The polarity in this lexicon ranged from -1 (the most negative connotation) to +1 (the most positive connotation).

### Analyses

Frequencies of both unigrams and bigrams were determined for each of the rating sentiment categories using the complete dataset and then by PM initiative and provider category. Output was further tailored by excluding unigrams and bigrams that did not provide insight into the reasoning behind the rating, including terms such as “bad,” “very good,” or “not satisfied.” This provided an overview of the most-used words or combination of words in each category. Next, the word polarity was averaged for all words in each PM initiative, which was compared to the average quantitative rating in the same initiative. The quantitative ratings have been studied in a previous study [14]. A linear regression analysis was added to determine if there was any correlation between the mean polarity and rating of each initiative.

In order to determine which words were the largest predictors of a positive, neutral, or negative rating, as determined by the NPS, supervised machine learning was used. Determining the most important predictors can provide insight into the reasoning of patients behind a rating: in other words, what patients value the most when providing a positive rating and what they dislike when they give a negative rating. The specific machine learning techniques used in this study are called naïve Bayes and random forest. The algorithms were run using the caret package in RStudio, version 1.1.383 (RStudio, Inc) [23]. Naïve Bayes is a fast method that performs well with a lot of dimensions and often performs similarly to other more complex methods [24]. A naïve Bayes model tries to predict, based on the words in a comment, the sentiment of a comment. It can be positive, neutral, or negative: the so-called classes. A naïve Bayesian classifier is based on the theorem of Bayes, in which predictors (ie, words) are assumed to be independent (ie, conditional independence); this theorem provides a method to calculate the posterior probability. The model uses this probability to predict the class (ie, sentiment). The dataset was randomly split between a training (50%) and a test (50%) dataset. The training set was used to train the models, while the test set was used to test the created models. Testing is done on an *unseen* set to prevent overfitting. The same test and training datasets were used in the random forest models. The algorithm creates multiple classification trees using a different bootstrap sample of the data. At each node of the tree, it chooses the best predictor out of a random subset of predictors [25]. The random forest model is known for its accuracy [26].Two models were run with each algorithm: one model aimed to predict the rating’s sentiment using the words in the comment; a second model tried to predict from which PM initiative a rating originated to determine if word use was different between initiatives. The goal was to see whether it was possible to identify unique strengths and/or weaknesses of PM initiatives. In the second model, the PM initiatives could be considered the classes. Using these outcomes, it was possible to determine the most important predictors of rating sentiment and of differences between initiatives, which could indicate what patients value or miss the most. The models were evaluated using the accuracy metric and confusion matrices. A confusion matrix shows how many predictions a model got right and wrong in each of the categories [27].

### Trial Registration

The Medical Research Involving Human Subjects Act (WMO) does not apply to this study, therefore, official approval was not required [28]. Participants agreed to the terms of service of Zorgkaart Nederland, which state that their submissions can be used anonymously for research purposes [29].

## Results

### Dataset

In total, 449,261 unsolicited ratings were available in the Zorgkaart database, coming from all providers in the Netherlands. These were given by 208,047 unique identifiers. Of these unsolicited ratings, 31,260 identifiers gave 70,800 ratings to providers in the PM initiatives (see Multimedia Appendix 1, Table A1.1). Of the 25,616 Dutch care providers that received at least a single rating in Zorgkaart, 4100 were located in one of the nine initiatives. The number of ratings within initiatives differed substantially, ranging from 1451 in the Vitaal Vechtdal region to 17,953 in the Slimmer met Zorg (SmZ) region (see Multimedia Appendix 1, Table A1.1). Each rating was accompanied by a comment.

### Sentiment

Based on the NPS, there were 303,930 positive ratings, 97,739 neutral ratings, and 47,592 negative ratings. This illustrates that patients were generally positive about the care they received. Unigrams did not give real insight into the reasoning behind ratings; words like “very,” “good,” “treatment,” and “satisfied” were very dominant. The unigrams are, therefore, not shown in the results. Before tailoring, many bigrams did not provide insight into the reasoning. For example, in the comments of neutral and positive ratings, most bigrams were related to general satisfaction with the service, for example, “very satisfied” and “very good.” Bigrams such as the following were, therefore, excluded: “very bad,” “very good,” “very satisfied,” “bad experience,” “good experience,” “helped well,” “very nice,” “takes all,” “not again,” “not good,” “totally not,” “just only,” “totally not,” “not really,” and “a lot.”

**Figure 1:**
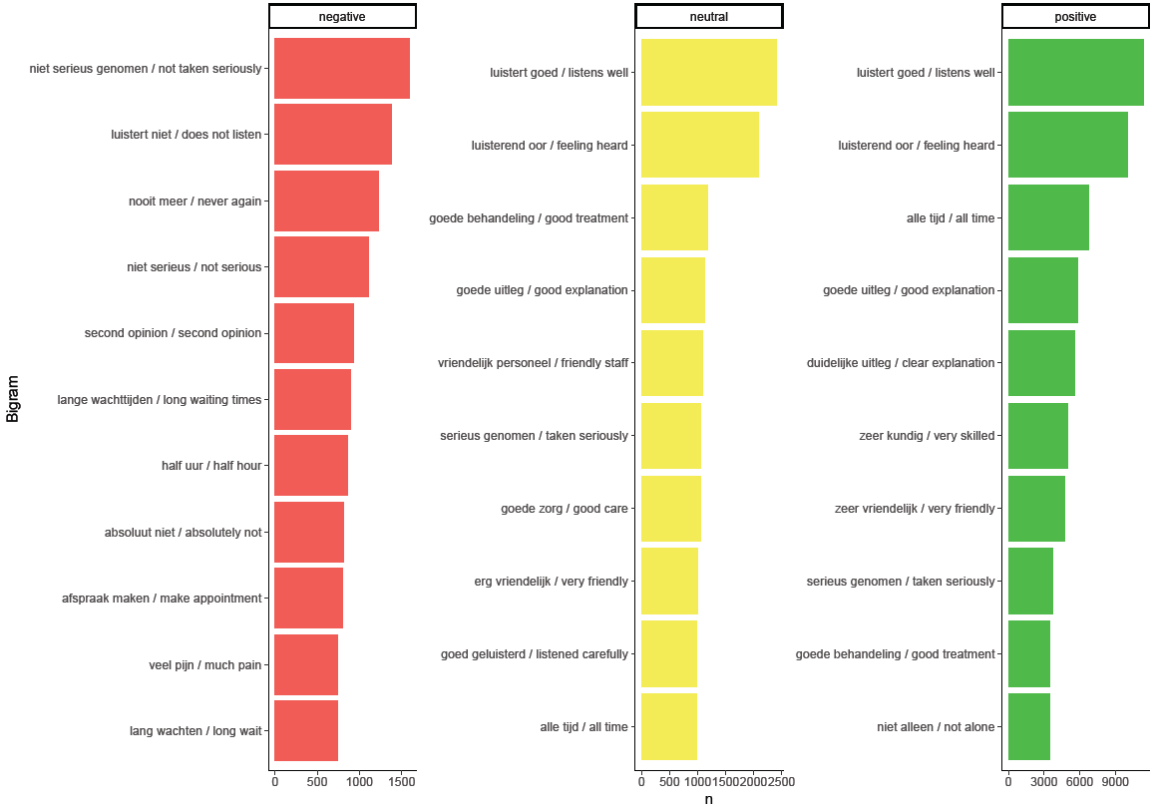
Most-used bigrams per the Net Promotor Score (NPS) category overall. The Dutch versions of the bigrams are listed to the left of the English translations.

The most-used bigrams after tailoring are shown in Figure 1. Negative bigrams were focused on listening and feeling like patients were being heard. The dominant term here was “not taken seriously.” Other bigrams within the negative ratings were mostly related to listening, waiting times, and not being satisfied with the treatment or diagnosis. Bigrams in the neutral and positive sentiment categories were similar and focused on being heard and kindness. These patterns were also seen when bigrams were split up by PM initiative (see Multimedia Appendix 2), with some exceptions. Positive ratings illustrated kindness in some and skill in other PM initiatives as the main topics, while negative ratings overall were mostly related to incorrect diagnoses and long waiting times. Standouts within the negative ratings include the region Gezonde Zorg, Gezonde Regio (ie, Healthy Care, Healthy Region), which mentioned a specific insurance company, and the mentioning of specific physicians by name in the Blauwe Zorg (ie, Blue Care) region.

When splitting up the dataset by provider type (see Multimedia Appendix 3), it becomes clear that different aspects mattered for different providers. For example, the amount of personnel was very important in nursing homes and was often considered in negative ratings, while the guidance by care providers was often considered a positive aspect of birth care.

**Figure 2:**
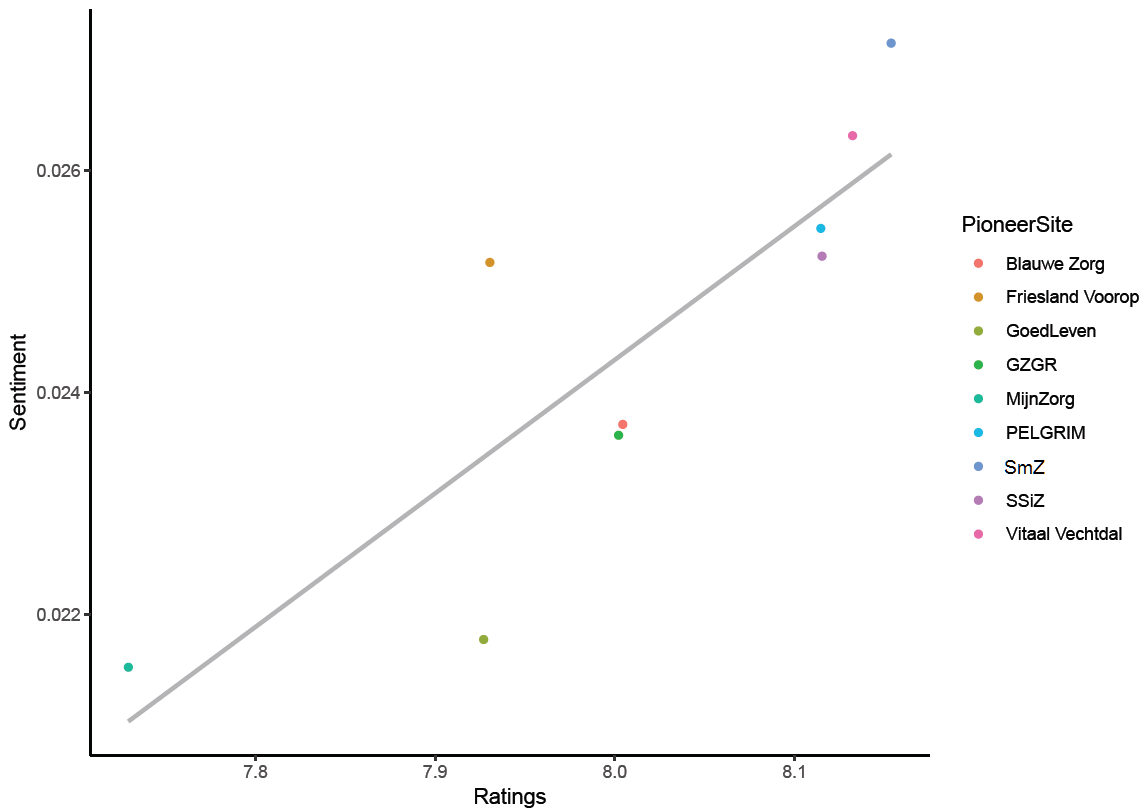
Correlation between ratings and sentiment with linear regression (r=.85): GZGR: Gezonde Zorg, Gezonde Regio; SSiZ: Samen Sterk in Zorg; SmZ: Slimmer met Zorg.

Both sentiment polarity and rating did not show a large range when averaged by PM initiative. Comparing the mean sentiment with the mean ratings showed a strong positive correlation (see Figure 2). A higher mean rating in a PM initiative indicated that the sentiment was actually better within that initiative.

### Machine Learning

Both the naïve Bayes and the random forest analyses were performed, but the results of the random forest are reported in the text, as these showed better results. The results of the naïve Bayes can be seen in Multimedia Appendix 4. Table 1 shows the confusion matrix of the sentiment model with the positive, neutral, and negative classes. The model was able to identify positive and negative ratings as such, but struggled with neutral ratings. Almost all neutral ratings were mistaken as positive ratings.

The words that had the biggest influence, including “satisfied,” “good,” “very,” and “fine,” were hard to interpret and were, therefore, not shown. Most words were simply similar to “good” or “bad,” but words related to employees seemed to be additional influential factors. They did not provide a clear indication of what patients value the most in each sentiment category. The results of the naïve Bayes analysis were similar (see Multimedia Appendix 4, Table A4.1). Bigrams were not tested as predictors, as their numbers were insufficient.

**Table 1:** Confusion matrix of sentiment analyses using random forest machine learning.

Prediction	Actual sentiment^a^, %		
	Negative	Neutral	Positive
Negative	49.2	9.7	2.7
Neutral	23.2	0.1	1.5
Positive	27.6	90.2	95.8
Total	100	100	100

^a^Accuracy=0.696.

Most ratings were positive, creating an imbalanced dataset. Additionally, the NPS scores we used could have influenced the results. Therefore, a sensitivity analysis was performed with a balanced dataset and only two sentiments: negative and positive. All ratings below 7.5 were considered to be negative, which yielded 98,974 results. An equal number of positive ratings were selected at random to create a balanced dataset. Other aspects of the analyses were kept identical to the previous analyses. This analysis showed that the accuracy improves drastically when using two sentiment categories, even when balancing them (see Table 2). The naïve Bayes variant of this analysis showed similar classifications but had worse accuracy (see Multimedia Appendix 4, Table A4.2).

**Table 2:** Confusion matrix of balanced sentiment analyses using random forest machine learning.

Prediction	Actual sentiment^a^, %	
	Negative	Positive
Negative	83.2	20.0
Positive	16.8	80.0
Total	100	100

^a^Accuracy=0.816.

The model attempting to predict PM initiatives based on comments was not as successful; the accuracy was low (0.26). Almost all ratings were classified as either PELGRIM or SmZ, which were the PM initiatives with the most ratings. However, nine categories are a lot to predict. To fine-tune the analysis, it was repeated with only the three-largest PM initiatives. The accuracy did increase (see Table 3), also due to the reduction in categories, but ratings were still mostly classified as PELGRIM and SmZ. This indicates that the model was not able to distinguish between the different PM initiatives and that the reasoning behind ratings was similar in each. Similar results were shown by the naïve Bayes analysis (see Multimedia Appendix 4, Table A4.3).

**Table 3:** Confusion matrix of the largest population management (PM) initiatives analyses using random forest machine learning.

Prediction	Actual PM initiative^a^, %		
	Friesland Voorop	PELGRIM	Slimmer met Zorg (SmZ)
Friesland Voorop	17.5	10.1	9.9
PELGRIM	30.6	41.4	27.2
Slimmer met Zorg (SmZ)	51.9	48.5	62.9
Total	100	100	100

^a^Accuracy=0.439.

## Discussion

### Principal Findings

This study explored the addition of comments accompanying unsolicited online ratings to regional analyses. The goal was to create additional insight for each PM initiative as well as for overall comparisons between initiatives by attempting to determine the reasoning and rationale behind a rating. A large online dataset provided by Zorgkaart Nederland, part of the DPF, was analyzed using sentiment analyses and machine learning techniques (naïve Bayes). Sentiment analyses illustrated that bigrams (ie, two-word combinations) proved to be more interpretable than unigrams (ie, single words). Feeling like not being “taken seriously” was the dominant bigram in negative ratings, while positive ratings mentioned mostly kindness and perceived knowledge. Comparing bigrams between PM initiatives showed a lot of overlap, but some small differences were present as well. When sentiments were quantified using a Dutch lexicon [22] and then by simply averaging the polarity of the words used, a strong correlation was found with the actual ratings. The machine learning models were able to identify sentiments of comments, especially the negative and positive comments. However, predictors did not give any meaningful insights into the underlying reasoning. When the second model tried to assign comments to PM initiatives, it could not distinguish between initiatives. This indicated that there was no clear difference in word use between initiatives.

The sentiment analyses showed that, when taken as a whole, the studied PM initiatives had mostly the same positive and negative aspects. Most ratings were positive and related to a kind and responsive staff, while negative ratings focused on being taken seriously, long waiting times, and misdiagnoses. These observations have been seen in the past in both solicited surveys [30] and interviews [31] and can be very useful for all PM initiatives, as they suggest that to get a positive rating, intangible aspects are important. Despite the amount of overlap between PM initiatives, some standout words are worth mentioning. For example, a specific care provider was mentioned often in negative ratings in a specific region. This very detailed information could prove to be very valuable for PM initiatives, as this could be used as a signal for further investigation.

### Limitations

The Zorgkaart data has to be interpreted with the inherent limitations of most online datasets in mind. The data are anonymous, making it impossible to correct for potential confounders, such as age, sex, and social economic status; thus, it is impossible to correct for selection bias. It is, for example, known that a younger, more tech-savvy population tends to provide online ratings [32]. Text analysis methods also often require vast amounts of data, which were not available for each of the studied initiatives. For example, this number of comments was insufficient for the use of trigrams (ie, three-word combinations). However, as the Zorgkaart dataset shows, it is growing faster each year and this issue should resolve itself over time. Additionally, it may be possible to combine text data from different sources to increase the amount of data. The machine learning results, combined with the polarity analyses, suggest that this is possible. For example, Twitter, Facebook, and Zorgkaart data in a region could be aggregated to strengthen sentiment analyses and comparisons.

### Future Research

As mentioned, online data have many benefits compared to other types of data. The biggest perk is probably the ability to monitor data close to real time. Policy makers and researchers often have to wait for survey or claims results regarding the output of an intervention; leveraging the strengths of online data could help here. In this study, the unsolicited online data have also shown that results in many regards are often similar to the results obtained from other sources. One next step for PM could, therefore, be to create a ratings dashboard for a region that keeps up with ratings and comments given in that region. It could show simple word clouds or frequencies or more advanced real-time results using machine learning; it should also be combined with more objective quality measures (eg, readmissions). This could give policy makers and researchers a more up-to-date idea of progress and might give them the opportunity to more quickly address any issues that could arise.

### Conclusions

Adding information from text comments that accompany online ratings to regional evaluations provides insight for PM initiatives into the underlying reasons for the ratings. Text comments provide useful overarching information for health care policy but due to a lot of overlap, there is only limited specific information for regional policy. Specific outliers for some initiatives are insightful but comparing PM initiatives remains difficult.

## Appendix

Multimedia Appendix 1 Description of Zorgkaart Nederland dataset.

Multimedia Appendix 2 Most-used words (ie, unigrams and bigrams) per population management (PM) initiative.

Multimedia Appendix 3 Most-used bigrams per provider category.

Multimedia Appendix 4 Additional results of the machine learning analyses.

## Abbreviations

DPF: Dutch Patient Federation
NPS: Net Promotor Score
PM: population management
SmZ: Slimmer met Zorg
WMO: Medical Research Involving Human Subjects Act
