# Supplementary material for: Measuring Regional Quality of Health Care Using Unsolicited Online Data: Text Analysis Study
Source: JMIR Med Inform. 2019 Dec 16;7(4):e13053. doi: 10.2196/13053 (PMC6937541; doi:10.2196/13053)
Supplement: Multimedia Appendix 1 [file medinform_v7i4e13053_app1.docx]

### Appendix 1. Description of *Zorgkaart Nederland* dataset


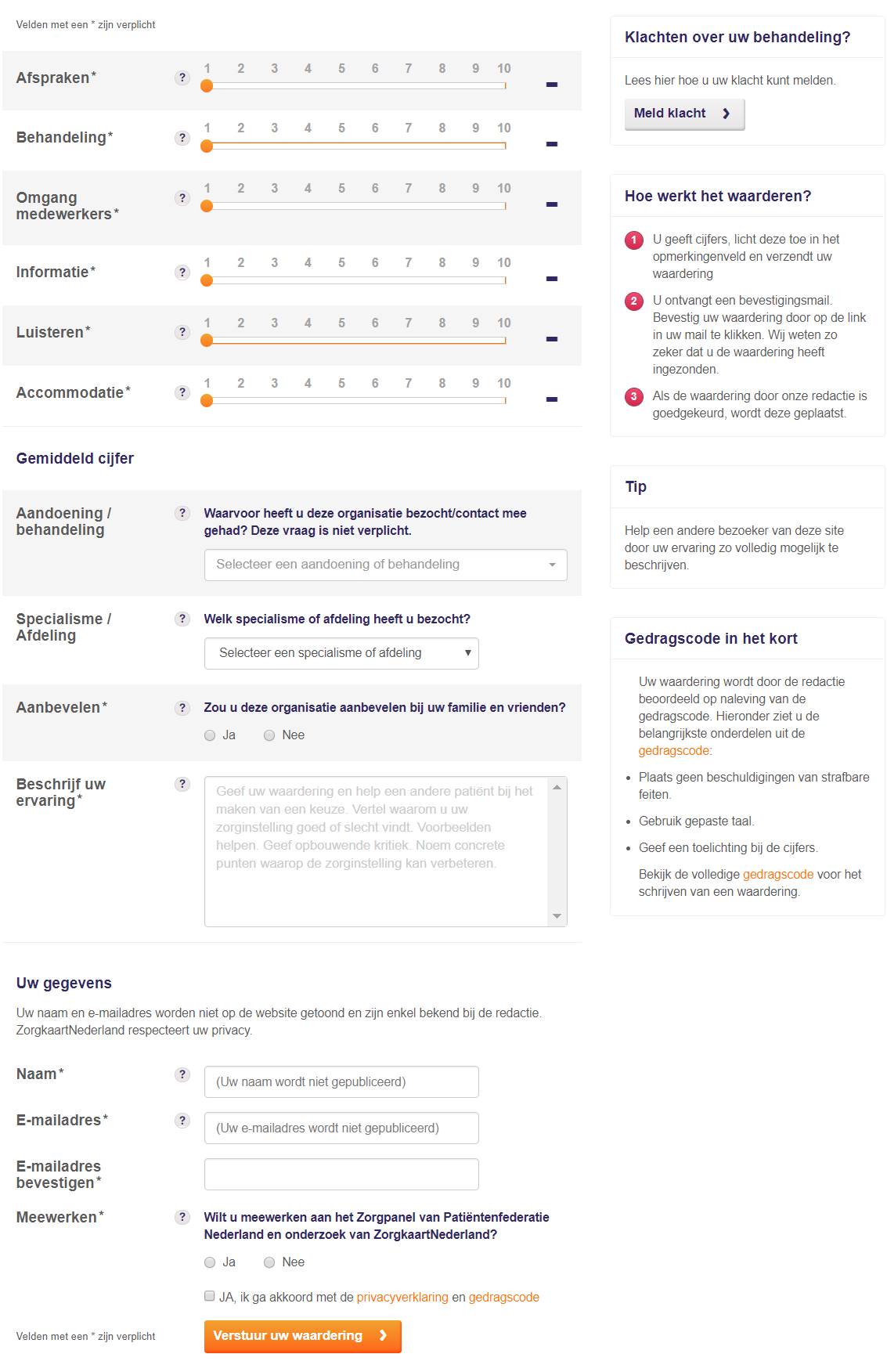


Figure A1.1. Screenshot of rating form Zorgkaart Nederland (Dutch)

Table A1.1. Number of ratings per provider category per PM initiatives

|  | Blauwe Zorg | Friesland Voorop | GoedLeven | MijnZorg | PELGRIM | GZGR | Smz | SSiZ | Vitaal Vechtdal | Total |
| --- | --- | --- | --- | --- | --- | --- | --- | --- | --- | --- |
| Birth care | 4 | 174 | 4 | 41 | 626 | 19 | 288 | 16 | 2 | 1174 |
| Dental care | 364 | 893 | 115 | 819 | 1577 | 667 | 1548 | 952 | 142 | 7077 |
| GP care | 918 | 1852 | 422 | 1543 | 1603 | 698 | 2590 | 1089 | 202 | 10917 |
| Home care | 170 | 131 | 31 | 119 | 540 | 25 | 377 | 72 | 35 | 1500 |
| Hospital care | 625 | 3150 | 901 | 1178 | 3745 | 1484 | 5645 | 1800 | 503 | 19031 |
| Insurers | 0 | 334 | 0 | 38 | 640 | 806 | 0 | 179 | 0 | 1997 |
| Nursing homes | 323 | 589 | 199 | 787 | 1158 | 290 | 1243 | 454 | 178 | 5221 |
| Other | 1024 | 3383 | 84 | 1230 | 4168 | 1379 | 4485 | 1868 | 104 | 17725 |
| Pharmacy | 106 | 168 | 44 | 113 | 345 | 77 | 214 | 237 | 7 | 1311 |
| Physiotherapy | 256 | 609 | 48 | 465 | 869 | 353 | 1556 | 384 | 278 | 4818 |
| Youth care | 0 | 15 | 0 | 3 | 3 | 0 | 7 | 1 | 0 | 29 |
| Total | 3790 | 11298 | 1848 | 6336 | 15274 | 5798 | 17953 | 7052 | 1451 | 70800 |
| Number of citizens | 176,055 | 646,910 | 106,270 | 273,500 | 417,780 | 183,920 | 516,500 | 273,340 | 112,655 | 270,6930 |
| Relative number of ratings (%) | 2,2 | 1,7 | 1,7 | 2,3 | 3,7 | 3,2 | 3,5 | 2,6 | 1,3 | - |

GZGR = Gezonde Zorg, Gezonde Regio; SSiZ = Samen Sterk in Zorg; SmZ = Slimmer met Zorg;

Table A1.2. Comparison and ANOVA of mean scores of online and survey data in nine PM initiatives (N=70,889)

|  | Online rating (SD) |
| --- | --- |
| Blauwe Zorg | 8.50 (1.82) |
| Friesland Voorop | 8.45 (1.89) |
| GoedLeven | 8.27 (1.92) |
| MijnZorg | 8.34 (1.96) |
| PELGRIM | 8.49 (1.69) |
| GZGR | 8.41 (1.84) |
| SMZ | 8.58 (1.71) |
| SSiZ | 8.50 (1.73) |
| Vitaal Vechtdal | 8.62 (1.65) |
| ANOVA | 0.000 |

GZGR = Gezonde Zorg, Gezonde Regio; SSiZ = Samen Sterk in Zorg; SmZ = Slimmer met Zorg;
