# Supplementary material for: Measuring Regional Quality of Health Care Using Unsolicited Online Data: Text Analysis Study
Source: JMIR Med Inform. 2019 Dec 16;7(4):e13053. doi: 10.2196/13053 (PMC6937541; doi:10.2196/13053)
Supplement: Multimedia Appendix 2 [file medinform_v7i4e13053_app2.docx]

### Appendix 2. Most used words (unigram/bigram) per PM initiative

Figure A2.1 Most used words (bigrams) in positive ratings per PM initiative

Figure A2.2. Most used words (bigrams) in negative ratings per PM initiative (*GoedLeven* and *Vitaal Vechtdal* were excluded due to the low amount of ratings)
