# Supplementary material for: Measuring Regional Quality of Health Care Using Unsolicited Online Data: Text Analysis Study
Source: JMIR Med Inform. 2019 Dec 16;7(4):e13053. doi: 10.2196/13053 (PMC6937541; doi:10.2196/13053)
Supplement: Multimedia Appendix 3 [file medinform_v7i4e13053_app3.docx]

### Appendix 3. Most used bigrams per provider category

Figure A3.1. Most used bigrams in positive ratings per provider category overall

Figure A3.2. Most used bigrams in negative ratings per provider category overall (translations are available in Appendix 2: “Translations”)
