# Supplementary material for: Measuring Regional Quality of Health Care Using Unsolicited Online Data: Text Analysis Study
Source: JMIR Med Inform. 2019 Dec 16;7(4):e13053. doi: 10.2196/13053 (PMC6937541; doi:10.2196/13053)
Supplement: Multimedia Appendix 4 [file medinform_v7i4e13053_app4.docx]

## Appendix 4. Additional results of machine learning analyses

Table A4.1. Confusion matrix of sentiment naïve Bayes machine learning

| Accuracy = 0.687 | | Actual sentiment | | |
| --- | --- | --- | --- | --- |
|  |  | Negative | Neutral | Positive |
| Prediction | Negative | 66,8% | 11,8% | 7,0% |
|  | Neutral | 12,5% | 37,0% | 9,1% |
|  | Positive | 20,7% | 51,2% | 83,9% |
|  | | 100% | 100% | 100% |

Table A4.2. Confusion matrix of balanced sentiment analyses using naïve Bayes machine learning

| Accuracy = 0.721 | | Actual sentiment | |
| --- | --- | --- | --- |
|  |  | Negative | Positive |
| Prediction | Negative | 60.47% | 16.21% |
|  | Positive | 39.53% | 83.79% |
|  | | 100% | 100% |

Table A4.3. Confusion matrix of largest population management initiatives naïve Bayes machine learning

| Accuracy = 0.399 | | Actual PM initiative | | |
| --- | --- | --- | --- | --- |
|  |  | Friesland Voorop | PELGRIM | SMZ |
| Prediction | Friesland Voorop | 0% | 0.02% | 0.02% |
|  | PELGRIM | 70.81% | 72.85% | 62.36% |
|  | SMZ | 29.19% | 27.13% | 37.62% |
|  | | 100% | 100% | 100% |
